# Simiao Wan and its ingredients alleviate type 2 diabetes mellitus *via* IRS1/AKT2/FOXO1/GLUT2 signaling

**DOI:** 10.3389/fnut.2022.1012961

**Published:** 2023-01-09

**Authors:** Ting Xia, Wen-Jie Xu, Yan-Nan Hu, Zhen-Ye Luo, Wen He, Chang-Shun Liu, Xiao-Mei Tan

**Affiliations:** ^1^School of Traditional Chinese Medicine, Southern Medical University, Guangzhou, China; ^2^Guangdong Provincial Key Laboratory of Chinese Medicine Pharmaceutics, Southern Medical University, Guangzhou, China; ^3^Guangdong Provincial Engineering Laboratory of Chinese Medicine Preparation Technology, Guangzhou, China; ^4^Guangdong Provincial Key Laboratory of Research and Development in Traditional Chinese Medicine, Guangzhou, China

**Keywords:** Simiao Wan, main bioactive ingredients, type 2 diabetes mellitus, network pharmacology, IRS1/AKT2/FOXO1/GLUT2 signaling

## Abstract

**Background:**

Type 2 diabetes mellitus (T2DM) is a metabolic disease. Simiao Wan (SMW) is a commonly used clinical drug for hyperuricemia treatment. SMW has been confirmed to improve insulin resistance and is expected to be a novel hypoglycemic agent. However, the hypoglycemic bioactive ingredients and mechanisms of action of SMW are unclear.

**Objective:**

To explore the hypoglycemic effects and reveal the mechanisms of SMW and bioactive ingredients (SMW-BI).

**Study design and methods:**

The hypoglycemic effects of SMW and SMW-BI were verified in a mouse model of T2DM induced by streptozotocin (STZ) and a high-fat and high-sugar diet (HFSD). Network pharmacology was used to predict the mechanisms of SMW and SMW-BI. Histological analysis and real-time quantitative polymerase chain reaction (RT-qPCR) verified network pharmacology results. RT-qPCR results were further verified by immunofluorescence (IFC) and molecular docking. The correlation between proteins and biochemical indicators was analyzed by Spearman’s correlation.

**Results:**

Chlorogenic acid, phellodendrine, magnoflorine, jateorhizine, palmatine, berberine, and atractydin were identified as SMW-BI. After 8 weeks of treatment, SMW and SMW-BI decreased the levels of fasting blood glucose (FBG), total cholesterol (TC), triacylglycerols (TG) and low-density lipoprotein cholesterol (LDL-C), increased the level of high-density lipoprotein cholesterol (HDL-C), alleviated weight loss, and increased serum insulin levels in T2DM mice. In addition, SMW and SMW-BI improved hepatocyte morphology in T2DM mice, decreased the number of adipocytes, and increased liver glycogen. Network pharmacological analysis indicated that SMW and SMW-BI may exert hypoglycemic by regulating insulin receptor substrate 1 (IRS1)/RAC-beta serine/threonine-protein kinase (AKT2)/forkhead box protein O1 (FOXO1)/glucose transporter type 2 (GLUT2) signaling. Moreover, correlation analysis showed that SMW and SMW-BI were associated with activation of IRS1, AKT2, and GLUT2, and inhibiting FOXO1. RT-qPCR revealed that SMW and SMW-BI could increase levels of IRS1, AKT2, and GLUT2 in the livers of T2DM mice and lower the level of FOXO1. Furthermore, immunofluorescence analysis showed that FOXO1 expression in the livers of T2DM mice decreased after oral administration of SMW and SMW-BI. Furthermore, molecular docking showed that SMW-BI could bind directly to IRS1 and AKT2.

**Conclusion:**

SMW and SMW-BI are potential hypoglycemic drugs that alleviate T2DM by regulating IRS1/AKT2/FOXO1 signaling. Our study provides a research idea for screening the bioactive ingredients in traditional Chinese medicine (TCM).

## 1. Introduction

Type 2 diabetes mellitus (T2DM) is a chronic inflammatory disease characterized by glycolipid metabolism disorders. Fasting blood glucose (FBG) ≥ 7.6 mmol/L, increased food intake, increased drinking water, and increased micturition are the clinical manifestations of T2DM (1). At present, 920,000 people worldwide have T2DM. There is a high prevalence of complex complications of T2DM such as diabetic nephropathy and diabetic cardiovascular disease, which seriously affect patients’ health and quality of life (2–4). Insulin injections and oral hypoglycemic drugs are effective treatments for T2DM. However, they are expensive and have serious gastrointestinal and cardiovascular side effects (5). T2DM is a systemic metabolic disease that does not rely on a single target drug. Therefore, identifying hypoglycemic drugs with good effects, low toxicity, and multiple targets has become an urgent clinical demand (6). Interestingly, the hypoglycemic effect of traditional Chinese medicine (TCM) formulas has been widely studied because of their lower price, fewer side effects, and multiple components and targets.

Simiao Wan (SMW) is a Chinese patent medicine widely used clinically. SMW consists of *Phellodendron amurense Rupr. (Rutaceae)* (PA) (voucher specimen: H0169121), *Atractylodes lancea (Thunb.) DC* (Asteraceae) (AL) (voucher specimen: C3120221), *Achyranthes bidentata Blume* (Amaranthaceae) (AB) (voucher specimen: N1469123), and *Coix lacryma-jobi L.* (Poaceae) (CL) (voucher specimen: Y4220414) at a 2:1:1:2 ratio. The plants’ part of SMW ingredient are the cortex of PA, rhizoma of AL, radix of AB, and seed of CL. The safety of SMW is guaranteed during its long-term clinical use. The insulin improvement effect of SMW has been confirmed in pharmacological studies. SMW can increase glycogen synthesis and inhibit liver triglycerides by mediating insulin production. SMW improves liver insulin sensitivity by regulating insulin receptor substrate 1 (IRS1) and AKT phosphorylation (7). In addition, SMW promotes glucose uptake by adipocytes by decreasing the phosphorylation of P65, inhibiting nuclear factor- κB (NF-κB) activation, and increasing adenosine 5′-monophosphate - activated protein kinase (AMPK) phosphorylation (8). However, there remains a lack of studies on the hypoglycemic effects of SMW, especially the bioactive ingredients of SMW (SMW-BI). Moreover, the hypoglycemic mechanisms of SMW and SMW-BI remain unclear.

Network pharmacology describes the interactions among compounds, targets, and diseases, which embodies the overall theory of TCM. In addition, network pharmacology can predict the bioactive ingredients and mechanisms of TCM for the treatment of diseases. Many studies have demonstrated the scientific nature of network pharmacology (9, 10). Network pharmacology provides a new research paradigm for evidence-based medicine systems and will accelerate our understanding of TCM.

Insulin resistance or deficiency is characteristic of T2DM and is known to impair glucose tolerance. The main manifestation of insulin resistance is the production of superfluous glucose in the liver. The liver has been proven to be one of the main target organs of hypoglycemic drugs. For example, Ramulus Mori (Sangzhi) alkaloids directly reduce FBG levels in obese mice by regulating liver and adipose tissue (11). Metformin (MET) is a classic clinical hypoglycemic drug that exerts a hypoglycemic effect in the liver by activating AMPK and regulating the inflammatory response in liver cells (12). Therefore, improving liver function and regulation of liver proteins is one of the main mechanisms of action of hypoglycemic drugs.

Our study aimed to explore the potential hypoglycemic effects of SMW and SMW-BI and to identify their hypoglycemic mechanisms. The content of bioactive ingredients in TCM is the basis of its efficacy. In this study, seven main bioactive ingredients (chlorogenic acid, phellodendrine, magnoflorine, jateorhizine, palmatine, berberine, and atractydin) with high content were screened by content determination analysis of SMW. The hypoglycemic effects of SMW and SMW-BI were confirmed in a mouse model of T2DM induced by a high-fat and high-sugar diet (HFSD) combined with streptozotocin (STZ). Network pharmacology was used to predict the hypoglycemic effects of SMW and SMW-BI, then the results were further verified by molecular biology experiments and docking.

## 2. Materials and methods

### 2.1. Materials and reagents

PA, AL, AB, and CL were purchased from Guangdong Traditional Chinese Medicine Co., Ltd. (Guangzhou, China). SMW-BI, including chlorogenic acid, phellodendrine, jateorhizine, magnoflorine, berberine, palmatine, and atractylodin, were obtained from the National Institutes for Food and Drug Control (Beijing, China). Acetonitrile was purchased from Merck (Darmstadt, Germany). STZ was purchased from Sigma-Aldrich (St. Louis, MO, USA). The insulin enzyme-linked immunosorbent assay (ELISA) kit was obtained from MEIMIAN Biotechnology Co., Ltd. (Jiangsu, China).

### 2.2. Preparation of SMW and SMW-BI

Simiao Wan was prepared by mixing 10 g PA, 20 g AL, 20 g CL, and 10 g AB in a round bottom flask, extracted for 1 h with 600 mL 95% ethanol, and filtered with 200 mesh gauze. Then, 600 mL deionized water was added to the residue and the extraction and filtration processes were repeated. The extract was concentrated at 50°C under a vacuum of 0.1 MPa. Finally, the concentrated solution was dried at −45°C and 80 Pa for 24 h to obtain freeze-dried SMW powder.

Phytochemical analysis of SMW was performed as previously described. Briefly, Agilent 1260 (Santa Clara, CA, USA) with diode-array detection was used. An Agilent ZORBAX SB-C18 (4.6 × 250 mm, 5 μm) was used to separate the SMW components. We used acetonitrile and 0.1% phosphoric acid as mobile phases for gradient elution. According to the results of a previous study, chlorogenic acid, phellodendrine, jateorhizine, magnoflorine, berberine, palmatine, and atractylodin were classified as SMW-BI. The contents of SMW-BI were as follows: chlorogenic acid, 0.72 mg/g; phellodendrine, 0.94 mg/g; magnoflorine, 0.089 mg/g; jateorhizine, 0.12 mg/g; palmatine, 0.16 mg/g; berberine, 8.48 mg/g; and atractydin, 0.76 mg/g.

**FIGURE 1:**
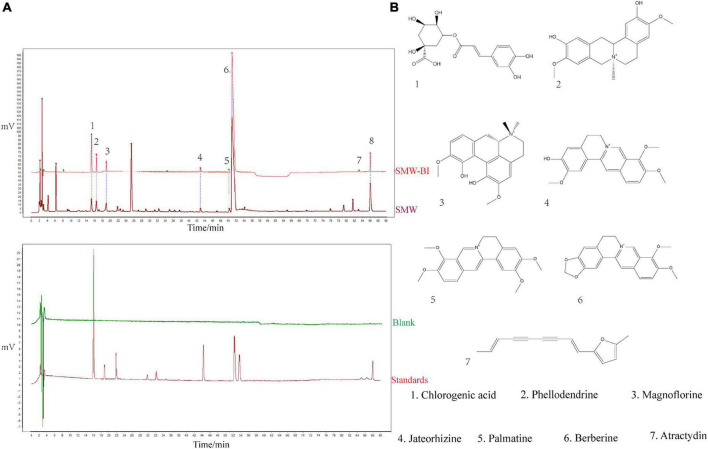
Phytochemical analysis of Simiao Wan (SMW) and bioactive ingredients of SMW (SMW-BI). Chromatogram of SMW-BI, SMW, blank, and standards (**A**, the concentration of SMW and standards are 40 mg/mL, 50 μg/mL). Chemical structures of SMW-BI **(B)**.

Bioactive ingredients of SMW (SMW-BI) was prepared based on the content and biological activity of the SMW components. HPLC was used to identify the components of SMW and SMW-BI (Figure 1).

### 2.3. Animals experiment

Forty, 5–6 weeks old, 22 ± 2 g male C57BL/6J were supplied by the Animal Center of Southern Medical University (Guangzhou, China). The number of animal license is SCXK(Yue)2016-0041). Mice were housed in an environment of 23 ± 1°C, 50–60% relative humidity, and a 12-h light/12-h dark cycle. The experimental protocols were performed in accordance with the Laboratory Animal Ethics Committee of the Southern Medical University.

Mice were randomly divided into five groups (*n* = 8) after 3 days adaption. The normal group (NC) was fed a normal chow diet, and the other mice were fed an HFSD (66.5 g/100 g normal chow diet, 20 g/100 g sucrose, 10 g/100 g lard oil, 2.5 g/100 g cholesterol, 1 g/100 g sodium cholate). After 4 weeks, The HFSD mice were intraperitoneally injected with 1% STZ (40 mg/kg) once every 2 days for three times. The STZ was dissolved in citrate buffer, at a concentration of 100 mM. FBG levels were detected by blood glucose monitoring (Sinocare, Changsha, China). Mice with an FBG concentration ≥ 11.1 mM and increased food intake, water consumption, urine output, and weight loss were selected for the subsequent experiments (13). All T2DM mice were fed an HFSD throughout the study.

Type 2 diabetes mellitus mice were randomly divided into a model group (MC), a MET group (200 mg/kg), an SMW group (1.2 g/kg), and an SMW-BI group (given the same content of SMW) (SMW-BI). MET, SMW, and SMW-BI were dispersed in normal saline. The treatment groups received oral gavage of MET, SMW, and SMW-BI once a day for 8 weeks. The NC and MC groups were fed saline every day.

### 2.4. Biochemical assays

#### 2.4.1. Determination of the levels of FBG and body weight

Fasting blood glucose levels were measured every 2 weeks from a vein using blood glucose monitoring after the start of oral administration. Before the determination of FBG, the mice fasted for 8 h. The body weights of mice during the study were determined every 2 weeks.

#### 2.4.2. Oral glucose tolerance test

On the last day of treatment, the oral glucose tolerance test (OGTT) was used to evaluate the ability of glucose metabolism of mice. Glucose was prepared in distilled water to 5 g/10 mL, oral glucose dose for mice was 2 g/kg. After glucose administration, we determined blood glucose levels at 0, 30, 60, and 120 min.

#### 2.4.3. Total cholesterol and triacylglycerol, low- and high-density lipoprotein cholesterol, and serum insulin determination

In order to completely metabolized the glucose we administrated, mice were sacrificed after 3 days of OGTT. Before the mice sacrificed, we used enzyme-free sterile tubes to collected blood samples. The blood samples were centrifuged at 4°C for 15 min (1250 × *g*), then the serum samples were obtained and stored at −80. The levels of total triacylglycerol (TG), total cholesterol (TC), high-density lipoprotein cholesterol (HDL-C) and low-density lipoprotein cholesterol (LDL-C) were measured by an automated biochemical analyzer (Shenzhen Leidu Life Science and Technology, Shenzhen, China). Serum insulin levels were measured using enzyme linked immunosorbent assay (ELISA) kits (Jiangsu Meimian industrial Co., Ltd., Yancheng, China).

### 2.5. Liver pathological staining and inflammation detection

After the mice were euthanized, liver samples were fixed in 4% paraformaldehyde (*n* = 8) and embedded in paraffin blocks. The blocks were cut into 5 μm sections and stained routinely with hematoxylin and eosin (H&E) and periodic acid-Schiff (PAS). Histopathological changes were observed using a light microscope.

IL-β, TNF-α, and IL-6 in mouse liver tissues were detected by ELISA kits. The liver samples were washed with PBS. After homogenized, the liver homogenates were centrifuged (1000 × *g*, 15 min) to obtain the supernatant. Then, we determined the protein concentration in the supernatant using a Total Protein Assay kit (Jiangsu Meimian industrial Co., Ltd., Yancheng, China). The supernatants were used to determine the concentrations of IL-β, TNF-α, and IL-6 using a commercial mice ELISA kit (Jiangsu Meimian industrial Co., Ltd., Yancheng, China).

### 2.6. Network pharmacological analysis

#### 2.6.1. Collection targets of SMW-BI and T2DM

The Swiss Target Prediction databases^1^ were used to collect the targets of the SMW-BI components. The key word “T2DM” was input into the Disgenet^2^ and Genecards^3^ databases to collect T2DM-related targets.

#### 2.6.2. Network construction

To further identify core targets for T2DM treatment, the targets were analyzed using the STRING database^4^, which currently has the largest number of organisms and proteins (14). Cytoscape 3.2.1 was used to construct the protein-protein interaction (PPI) and component-target-collateral (C-T-C) network (15).

#### 2.6.3. Gene Ontology and Kyoto Encyclopedia of Genes and Genomes pathway enrichment analysis

Gene Ontology (GO) enrichment analysis was performed to further study the cellular components (CC), biological processes (BP) and molecular functions (MF) of the identified potential anti-T2DM target genes of SMW-BI based on the DAVID database.^5^ The main potential biological pathways were analyzed by Kyoto Encyclopedia of Genes and Genomes (KEGG) pathway enrichment analysis. Functional terms and pathways were visually analyzed using R 4.0.2 (R Foundation, Vienna, Austria) (16).

### 2.7. Real-time quantitative PCR

After the mice were sacrificed, liver samples were obtained, immediately quenched in liquid nitrogen, and stored at −80°C. Liver samples were removed before real-time quantitative polymerase chain reaction (RT-qPCR) analysis. RT-qPCR was carried out as previously described (17). After total RNA extraction, cDNA reverse transcription, and primer amplification, gene expression was analyzed. The corresponding primer sequences are listed in Table 1. The relative expression was calculated using the 2^–△△*Ct*^ method.

**TABLE 1:** Sequences of primers used for real-time quantitative polymerase chain reaction (RT-qPCR).

Genes	Forward (5′ to 3′)	Reverse (3′ to 5′)	Size (bp)	Annealing temperature (°C)
AKT2	GGCAAGGTCATTCTGGTTCGA	GCATAGGCGGTCATGGGTCT	189	60
FOXO1	GAGTGGATGGTGAAGAGCGTG	AAGGGACAGATTGTGGCGAA	96	60
GLUT2	GAGCCCTCTTGATGGGATGTT	AAGGGCCAGTTGGTGAAGAGTAC	176	60
IRS1	CATCTCAACAACCCTCCACCC	GGTTTCCCACCCACCATACTG	101	60
GAPDH	CCTCGTCCCGTAGACAAAATG	TGAGGTCAATGAAGGGGTCGT	133	60

### 2.8. Immunohistochemistry and immunofluorescence

Immunohistochemistry (IHC) was used to analyze the expression of IRS1, AKT2, and glucose transporter type 2 (GLUT2) in the mouse livers. Specifically, the wax-embedded livers were cut into 5 μm sections. We used anti-mouse IRS1 (Servicebio, Wuhan, China), AKT2 (Proteintech, Rosemont, IL), and GLUT2 (Servicebio, Wuhan, China) antibody (1:100) at 4°C overnight for IHC staining. All slices were counterstained with hematoxylin at 25°C for 1 min. Microscopic images were obtained with a light microscope. Five random areas from each sample were chosen and analyzed using Image J (National Institutes of Health, Bethesda, MD, USA).

Immunofluorescence (IFC) was carried out as previously described (18). Wax-embedded livers were cut into 5 μm slices, and then the samples were dewaxed with dewaxing solution, ethanol, and distilled water. EDTA antigen buffer was used to repair antigens. Then, samples were incubated with bovine serum albumin for 30 min to block the antigen. Samples were incubated with forkhead box protein O1 (FOXO1) primary antibody (dilution 1:700; Servicebio, Wuhan, China) at 4°C for 24 h, followed by FITC-conjugated secondary antibody (dilution 1:1000; Servicebio, Wuhan, China) at 25°C for 50 min. DAPI was used to dye the nucleus at 25°C in the dark for 10 min. The fluorescence quenching agent was added and samples were analyzed under a fluorescence inversion microscope (ECLIPSE-TE 2000; Nikon, Tokyo, Japan).

### 2.9. Molecular docking

To further verify the RT-qPCR results, SMW-BI was docked with AKT2 and IRS1. Crystal structures of IRS1 (ID:1K3A) and AKT2 (ID:1O6K) were obtained from the PDB database.^6^ Discovery Studio 2019 (Dassault Systèmes, Vélizy-Villacoublay, France) was used to conduct follow-up docking experiments. Briefly, the proteins were dehydrated, hydrogenated, and added to electric fields to form an active pocket. Then, molecular docking between proteins and molecular compounds was performed using the default setting. The binding forces between the small molecules and proteins were analyzed using the docking fraction (19).

### 2.10. Statistical analysis

Experimental data was analyzed with GraphPad Prism 8.3 (GraphPad Software, San Diego, CA). One-way analysis of variance was used to compare data between groups. All data are expressed as mean ± standard deviation (SD). Statistical significance was set at *p* < 0.05 (*p < 0.05, ^**^p < 0.01).

## 3. Results

### 3.1. Biochemical assays

#### 3.1.1. SMW and SMW-BI decreased FBG and prevented weight loss in T2DM mice

**FIGURE 2:**
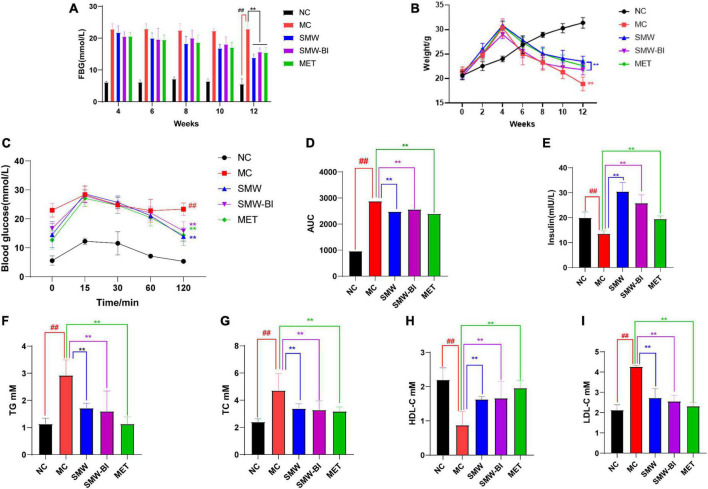
Effects of Simiao Wan (SMW) and bioactive ingredients of SMW (SMW-BI) on FBG **(A)**, body weight **(B)**, oral glucose tolerance (OGTT) **(C)**, AUC0-2h **(D)**, serum insulin **(E)**, serum total triacylglycerol (TG) **(F)**, total cholesterol (TC) **(G)**, high density lipoprotein cholesterol (HDL-C) **(H)**, and low-density lipoprotein cholesterol (LDL-C) **(I)** data are presented as the mean ± SD (*n* = 8). **P < 0.01 versus the model group (MC group), ^##^*p* < 0.01 versus the normal group (NC group).

Fasting blood glucose levels in each group were measured every 2 weeks after the 4th week. After injection of STZ in the 4th week, the FBG level of the HFSD mice was higher than that of the normal group (NC group), which was higher than 11.1 mmol/L. After oral administration of SMW, SMW-BI, and MET for 8 weeks, FBG levels in the treatment groups were significantly decreased compared to those in the MC group (Figure 2A). In addition, before STZ injection, the weight of mice in the HFSD group increased faster than that of the NC group. However, the weight of T2DM mice decreased sharply after STZ injection. After intragastric administration of SMW, SMW-BI, and MET for 8 weeks, weight loss in the treatment group was lower than that in the MC group (Figure 2B). The results revealed that SMW and SMW-BI can prevent weight loss caused by T2DM, similar to MET.

#### 3.1.2. SMW and SMW-BI improved the OGTT and serum insulin of T2DM mice

Oral glucose tolerance test results showed that the blood glucose reached a maximum after oral administration of glucose for 15–30 min, and then decreased gradually with time. After 120 min of oral glucose administration, the blood glucose of the mice reached a steady state. Compared with the NC group, the rate of glycemic fall in the MC group was slower, indicating that T2DM mice had more difficulty in degrading glucose. Interestingly, the blood glucose levels of the SMW, SMW-BI, and MET groups decreased significantly compared to the MC group (Figure 2C). The area under the curve from 0 to 2 h (AUC_0–2__*h*_) after oral glucose administration was used to analyze the glucose tolerance of each group of mice. SMW and SMW-BI significantly improved glucose tolerance in mice, but there was no significant difference between SMW and MET or SMW-BI and MET (Figure 2D).

We investigated the effects of SMW and SMW-BI on levels of serum insulin in T2DM mice. Serum insulin levels in the NC group were significantly higher than those in the MC group. The SMW, SMW-BI, and MET groups showed higher serum insulin levels after oral administration (Figure 2E).

#### 3.1.3. SMW and SMW-BI improved blood lipids in T2DM mice

To explore the effects of SMW and SMW-BI on blood lipid levels in T2DM mice, we measured TG, TC, LDL-C, and HDL-C in the T2DM mice. The level of HDL-C was significantly lower, while LDL-C, TG, and TC in the MC group were significantly higher than those in the NC group. After treatment with SMW, SMW-BI, and MET for 8 weeks, the levels of TC, LDL-C, and TG decreased, and the level of HDL-C increased significantly (Figures 2F–I). These results suggest that SMW and SMW-BI can improve the blood lipid levels in T2DM mice.

### 3.2. SMW and SMW-BI improved liver cell morphology, increased liver glycogen, and decreased liver inflammation

**FIGURE 3:**
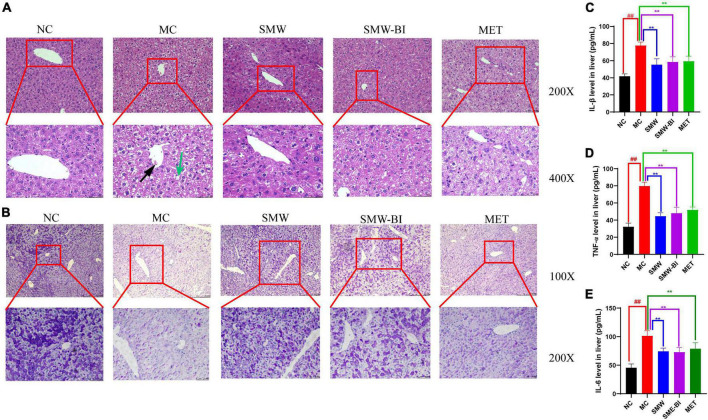
Histological analysis of liver tissue specimens. Hematoxylin and eosin (H and E) staining of liver tissues **(A)**, periodic acid-schiff (PAS) staining of liver tissues **(B)**. The expression of IL-β **(C)**, TNF-α **(D)**, and IL-6 **(E)** in mouse livers. Black arrows indicate inflammatory cell infiltration and green arrows indicate degenerating adipocytes in the mouse liver tissue sections. The red squares which extending out were local magnifications. Data are present as the mean ± SD (*n* = 8). **P < 0.01 versus the model group (MC group), *^##^p* < 0.01 versus the normal group (NC group).

Liver cell injury is a common problem in diabetes (20). To evaluate the effects of SMW and SMW-BI on hepatocytes of T2DM mice, liver samples were stained with H and E. Compared to the NC group, the arrangement of hepatocytes in the MC group was disordered, and the cell boundary was not clear. In addition, in the MC group, liver fat cell degeneration (green arrow) was significantly reversed by SMW, SMW-BI, and MET treatment (Figure 3A). Moreover, SMW and SMW-BI also improved inflammatory infiltration in the liver of T2DM mice (black arrow).

Disturbance in hepatic glycogen synthesis is another pathological feature of diabetes (21). PAS staining was used to explore the effects of SMW and SMW-BI on hepatic glycogen in T2DM mice. Glycogen in the liver samples was stained purple-red and located in the cytoplasm. Glycogen density in the NC group was higher than that in the MC group, and the distribution was uneven. After treatment with SMW and SMW-BI for 8 weeks, the hepatic glycogen density was significantly higher than that in the MC group (Figure 3B).

The increase of inflammatory factors in the liver is one of the manifestations of liver injury. Our results indicated that SMW and SMW-BI decreased the level of IL-β, TNF-α, and IL-6 in T2DM mice livers (Figures 3C–E).

### 3.3. Network pharmacology

**FIGURE 4:**
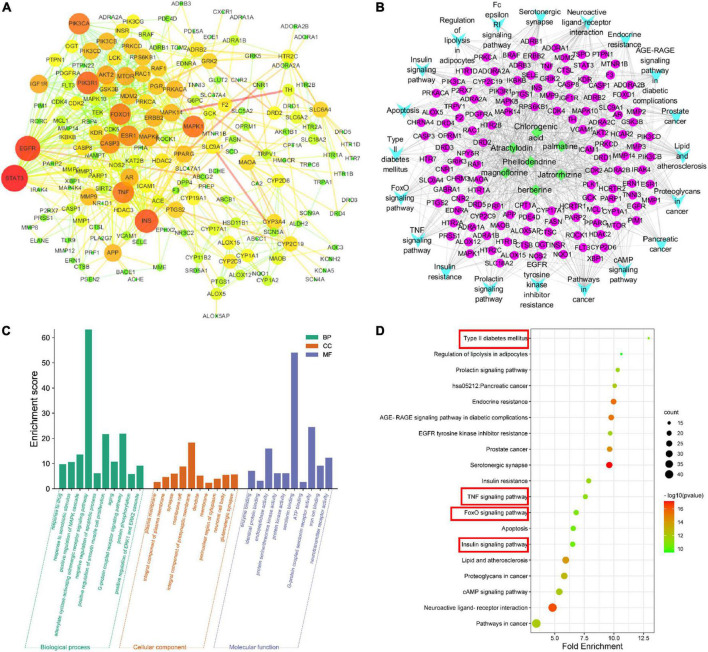
Network pharmacology analysis. protein-protein interaction (PPI) network **(A)**, component-target-collateral (C-T-C) network **(B)**, Gene Ontology (GO) analysis **(C)**, and Kyoto Encyclopedia of Genes and Genomes (KEGG) analysis **(D)**.

The PPI results show interactions between proteins, where the size of a circle represents the strength of the protein interaction. INS, PIK3R1, FOXO1, and AKT2 showed strong interactions (Figure 4A and Table 2), indicating these proteins play key roles in the hypoglycemic effect of SMW and SMW-BI.

**TABLE 2:** The degree of key genes for type 2 diabetes mellitus (T2DM).

Genes	Degree	Radiality
STAT3	40	0.8198
EGFR	36	0.8293
INS	32	0.8150
PIK3R1	28	0.7761
MAPK1	27	0.8048
FOXO1	27	0.7936
PIK3CA	27	0.7698
TNF	25	0.8055
ESR1	24	0.7920
CASP3	21	0.7992
AKT2	21	0.7634
MAPK8	20	0.7888
IGF1R	20	0.7698
MTOR	19	0.7730
AR	19	0.7801

To further analyze the mechanism of SMW and SMW-BI in the treatment of T2DM, we performed GO analysis of the key targets. The molecular mechanism is mainly related to BP, CC, and MF. BP mainly included negative regulation of the apoptotic process, positive regulation of the MAPK cascade, G-protein coupled receptor signaling pathway, and adenylate cyclase-activating adrenergic receptor signaling pathway. CC included the integral components of the plasma membrane, composition, and synapse. MF included endopeptidase activity, protein binding and enzyme binding (Figure 4C). KEGG pathway analysis was used to explore the hypoglycemic pathways of SMW and SMW-BI. The KEGG analysis results showed that the main hypoglycemic pathways were the insulin, T2DM, TNF, and FOXO signaling pathways (Figures 4B, D).

### 3.4. SMW and SMW-BI regulated the insulin receptor substrate 1 (IRS1)/RAC-beta serine/threonine-protein kinase (AKT2)/forkhead box protein O1 (FOXO1)/glucose transporter type 2 (GLUT2) axis in the T2DM mouse liver

**FIGURE 5:**
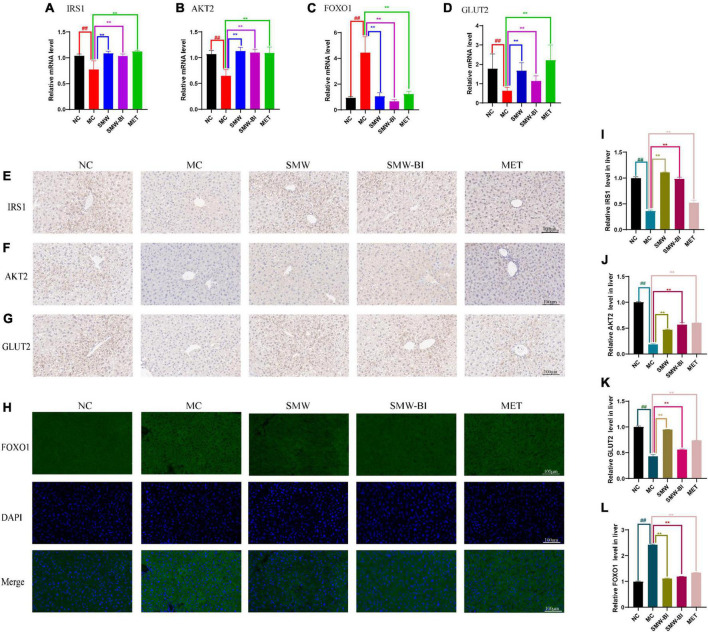
Effects of Simiao Wan (SMW) and bioactive ingredients of SMW (SMW-BI) on insulin receptor substrate 1 (IRS1)/RAC-beta serine/threonine-protein kinase (AKT2)/forkhead box protein O1 (FOXO1)/glucose transporter type 2 (GLUT2) signaling in type 2 diabetes mellitus (T2DM) mice. Levels of mRNA expression of Irs1, Akt2, Foxo1 and Glut2 **(A–D)**. IHC of IRS1 **(E,I)**, AKT2 **(F,J)**, and GLUT2 **(G,K)**. IFC of FOXO1 **(H,L)**. Data are presented as the mean ± SD (*n* = 8). **P < 0.01 versus the model group (MC group), *^##^p* < 0.01 versus the normal group (NC group).

We used RT-qPCR to verify the expression of *Irs1*, *Akt2*, *Foxo1*, and *Slc2a2* (which encodes GLUT2), which are involved in the insulin and FOXO signaling pathways. RT-qPCR results showed that *Irs1*, *Akt2*, *Slc2a2* decreased while *Foxo1* increased significantly in T2DM mice compared with the NC group. After oral administration of SMW and SMW-BI, the expression levels of these genes approached the levels in the NC group (Figures 5A–D). The results of IHC and IFC also suggested that SMW and SMW-BI increase the levels of IRS1, AKT2, and GLUT2, while lowering FOXO1 (Figures 5E–L). FOXO1 mediates the expression of downstream GLUT2, which is beneficial for glucose transport (22). The above results indicate that SMW and SMW-BI can activate IRS1, AKT2, and GLUT2, while inhibiting FOXO1 in T2DM hepatocytes. The reliability of the network pharmacology results was verified.

### 3.5. SMW-BI bind directly to IRS1 and AKT2

**FIGURE 6:**
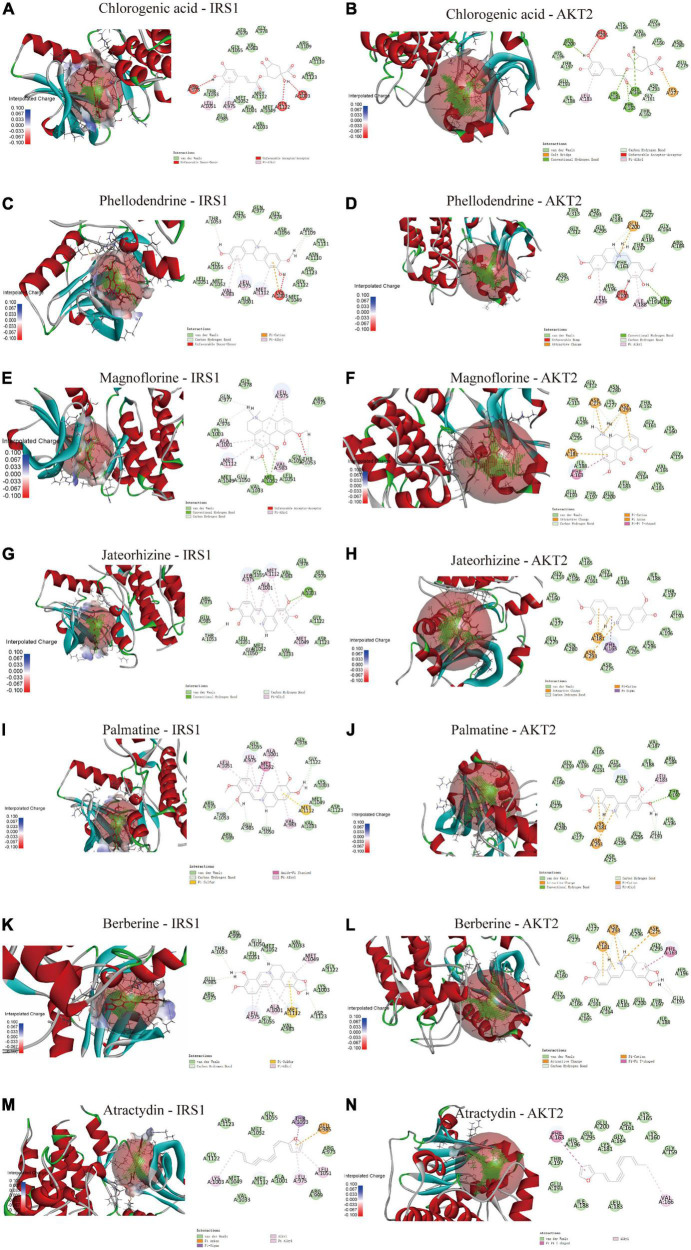
The molecular docking of SMW-BI with insulin receptor substrate 1 (IRS1) and RAC-beta serine/threonine-protein kinase (AKT2) **(A–N)**.

Molecular docking was used to determine the binding ability of SMW-BI to proteins. The components of SMW-BI were docked with IRS1 and AKT2, and the methodology was verified using protein ligands. Molecular docking showed that SMW-BI can bind directly to IRS and AKT2 (Table 3). Among them, chlorogenic acid, jateorhizine, and phellodendron had the strongest binding ability to proteins. Docking results showed that SMW-BI binds to IRS1 and AKT2 mainly through carbon hydrogen bonds and van der Waals forces (Figures 6A–N). Molecular docking verified the results of the molecular experiments.

**TABLE 3:** Docking scores of bioactive ingredients of SMW (SMW-BI) with insulin receptor substrate 1 (IRS1) and RAC-beta serine/threonine-protein kinase (AKT2).

	Ligands RMSD (≤2)	Chlorogenic acid	Phellodendrine	Magnoflorine	Jateorhizine	Palmatine	Berberine	Atractydin
IRS1	1.6505	96.3584	93.3654	87.3707	96.1945	91.2872	92.0867	64.6741
AKT2	1.5707	126.0580	121.8040	93.5972	107.9010	103.7410	101.0050	75.8706

RMSD, root-mean-square deviation.

### 3.6. Spearman’s correlation analysis

Spearman’s rank correlation analysis (false discovery rate < 0.05) was carried out to investigate whether the hypoglycemic effects of SMW and SMW-BI were related to the expression of the IRS1/AKT2/FOXO1/GLUT2 signaling pathway.

**FIGURE 7:**
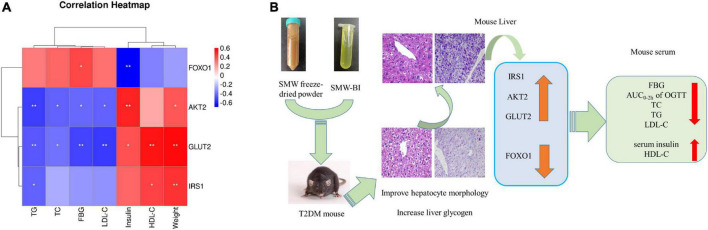
Spearman’s analysis of hypoglycemic effect and genes **(A)**. Possible mechanism underlining the Simiao Wan (SMW) and bioactive ingredients of SMW (SMW-BI) treatment of type 2 diabetes mellitus (T2DM) in mice **(B)**. Red represents positive correlation, and blue indicates negative correlation. *Represents a significant correlation between biochemical indicators and **represents an extremely significant correlation between biochemical indicators.

As shown in Figure 7A, IRS1 and GLUT2 were positively associated with HDL-C levels and mouse weight, AKT2 was positively associated with insulin levels, and FOXO1 was negatively correlated with FBG. GLUT2 and AKT2 levels were negatively associated with TC, TG, LDL-C, and FBG. These results suggest that the hypoglycemic effects of SMW and SMW-BI are related to the IRS1/AKT2/FOXO1/GLUT2 signaling pathway.

## 4. Discussion

In this study, seven bioactive ingredients at high contents in SMW were used as SMW-BI, and the hypoglycemic effects of SMW and SMW-BI were evaluated in a mouse model of T2DM induced by HFSD combined with STZ. Our results showed that SMW and SMW-BI can reduce TG, FBG, TC, and LDL-C levels and improve glucose tolerance, serum insulin, HDL-C, hepatocyte morphology, and liver glycogen synthesis in T2DM mice. These physiological indices are similar to the effects of MET.

Metformin is used as a first-line hypoglycemic treatment for T2DM patients since it was recommended by the European Association for the Study of Diabetes (EASD) and the American Diabetes Association (ADA) (23). MET improves hyperglycemia by suppressing hepatic gluconeogenesis, decreasing hepatic glucose output, elevating glucose uptake and utilization in peripheral tissues, and enhancing the energy metabolism in several organs, such as muscle, fat, and liver through the activation of AMPK (24). However, there has been widespread concern about the adverse effects of MET during long-term clinical use. The gastrointestinal tract is one of the sites affected, with side effects including nausea, vomiting, and diarrhea (25). In order to observe the side effects of SWM, SMW-BI, and MET, we measured the viscera index of gastric and spleen in each group mice. The viscera indexes of the gastric and spleen in the MC group was higher than that in the NC group. After oral administration of SMW and SMW-BI, the gastric and spleen organ indices were improved. However, in the MET group, the gastric and spleen organ indices were higher than those in the MC group (Table 4). More importantly, the body weight of the SMW group was better than that of the MET group, and we observed that autonomic activity was more frequent in the SMW and SMW-BI groups than in the MET group. The results revealed that the hypoglycemic effect of SMW and SMW-BI was similar to that of MET, whereas the side effects were less severe than those of MET.

**TABLE 4:** Effect of Simiao Wan (SMW) and bioactive ingredients of SMW (SMW-BI) on stomach and spleen index in mice.

Groups	Gastric index (%)	Spleen index (%)
NC	1.20 ± 0.11^a^	0.20 ± 0.06^a^
MC	2.01 ± 0.14^b^	0.41 ± 0.03^b^
SMW	1.27 ± 0.08^a^	0.25 ± 0.02^a^
SMW-BI	1.31 ± 0.05^a^	0.24 ± 0.04^a^
MET	3.85 ± 0.13^c^	1.08 ± 0.03^c^

Data are present as the mean ± SD (*n* = 8). Bars marked without same letters (a–c) differ significantly (*p* < 0.05).

The insulin-mediated IRS1/AKT/FOXO1 signaling pathway is closely related to pancreatic β-cell function, liver glucose metabolism, and the occurrence and development of T2DM (26). In recent years, the IRS1/AKT/FOXO1 signaling pathway has attracted wide attention as a key link in the pathogenesis and treatment of T2DM. Interestingly, the network pharmacology results also suggested that SMW and SMW-BI exerted hypoglycemic effects through the IRS1/AKT2/FOXO1 signaling pathway. IRS1 is a key receptor of the insulin signaling pathway, which maintains the basic functions of cell growth, division, and metabolism (27). IRS1, a member of the IRS protein family and the main substrate of IGF1R, participates in insulin regulation. Our results also verified the above theory: when FBG levels in T2DM mice decreased, serum insulin levels increased, and IRS1 expression in the liver also significantly increased. In addition, SMW-BI directly binds to IRS1, further indicating that SMW and SMW-BI can regulate IRS1. SLC2A2, AKT, and the PI3K gene family are key targets in the insulin signaling pathway and play important roles in activating insulin and liver glucose transport (28). Network pharmacology showed that GLUT2, PI3K, and AKT2 were the key targets of SMW and SMW-BI in glucose lowering. RT-qPCR showed that SMW and SMW-BI significantly increased the expression of GLUT2 and AKT2 in the liver. In addition, molecular docking indicated that SMW-BI had a good binding ability to AKT2, which further verified that the hypoglycemic pathway of SMW and SMW-BI was related to AKT2. FOXO1 is involved during lipid metabolism and the occurrence and development of T2DM (29, 30). Furthermore, FOXO1 is one of the most important transcription factors in insulin synthesis (31) and is widely distributed in the spleen, liver, and visceral adipose tissue, mainly coordinating the differentiation of adipocytes (32). It is widely accepted that AKT/FOXO1 signaling is the core intermediary in liver glycogen production (33), which is consistent with our results from network pharmacology, RT-qPCR, and immunofluorescence staining. FOXO1 binds to the promoters of PEPCK and G6Pase through its fork-box DNA-binding domain, thereby promoting its expression (34). Our study revealed that SMW and SMW-BI inhibited the level of FOXO1 *via* strong expression of the AKT pathway. FOXO1 inhibition not only reduces the excessive production of liver glycogen but also improves the degeneration of adipocytes due to the increased utilization of glucose, thus reducing blood lipids (35). In our study, the blood lipid indices of T2DM mice were significantly improved. Spearman’s correlation analysis revealed that FBG, insulin, and HDL-C in T2DM mice were positively correlated with IRS1, AKT2, and GLUT2 and negatively correlated with FOXO1.

During the animal experiments, we converted the clinical human dose of SMW into a mouse dose for pharmacodynamic verification. We did not consider higher or lower doses because they are rarely used clinically. Chlorogenic acid, phellodendrine, jateorhizine, magnoflorine, berberine, palmatine, and atractylodin are the components of SMW-BI, according to the screening principle of high content and strong biological activity. Network pharmacology predicted that SMW and SMW-BI exerted hypoglycemic effects *via* IRS1/AKT/FOXO1 signaling. Many ingredients in SMW-BI have been reported to lower blood sugar levels. For example, chlorogenic acid can effectively prevent the development of T2DM induced by HFSD combined with STZ, which is related to the inhibition of G-6-Pase mRNA in the liver and upregulation of GLUT4 mRNA in the skeletal muscle (36). Magnoflorine increases AKT phosphorylation, inhibits autophagy and proteolysis, and decreases blood glucose levels in T2DM rats (37). Islet cell dysfunction is a key factor in the pathogenesis of T2DM (38). Previous studies have shown that berberine alleviates β-cell dysfunction by regulating the mir-204/SIRT1 pathway (39). Berberine, as the most abundant ingredient in SMW and SMW-BI, has a strong hypoglycemic effect, including increasing the expression of insulin receptors, reducing blood sugar (40), and relieving the intestinal mucosal barrier in T2DM (41). Berberine also exerts pharmacological effects through the intestine because of its low oral bioavailability, such as regulating the structure of the gut microbiota, improving the intestinal barrier, and reducing blood glucose (42, 43). Jatrorrhizine, palmatine, and berberine extracted from Coptis chinensis can be used as α-glucosidase inhibitors (44). Consistent with the results of many studies, our results showed that SMW-BI effectively reduced blood glucose levels in T2DM mice. This further illustrates the accuracy of the screening method for the bioactive ingredients selected in the study.

However, the FBG of the SMW-BI group was higher than that of the SMW group. These results suggest that there are other components in SMW besides SMW-BI that can exert hypoglycemic effects. For example, according to previous studies, we have shown that coix seed polysaccharides in SMW can exert a hypoglycemic effect by regulating the gut microbiota (17).

## 5. Conclusion

In this study, *in vivo* experiments and network pharmacology, combined with molecular biology experiments and molecular docking, indicated the potential pharmacological mechanism of the hypoglycemic effect of SMW and SMW-BI. Our results showed that SMW and SMW-BI can alleviate T2DM by lowering FBG levels and increasing serum insulin and hepatic glycogen synthesis, and the mechanism is related to the regulation of the IRS1/AKT2/FOXO1/GLUT2 pathway. After treatment with SMW, SMW-BI, and MET for 8 weeks, the levels of TC, LDL-C, and TG decreased, and the level of HDL-C increased significantly (Figure 7B). This study provides a theoretical basis for the clinical application of hypoglycemic SMW and a research method for screening the bioactive ingredients of TCM.

## Data availability statement

The raw data supporting the conclusions of this article will be made available by the authors, without undue reservation.

## Ethics statement

This animal study was reviewed and approved by the Laboratory Animal Ethics Committee of the Southern Medical University.

## Author contributions

TX performed the experiment and wrote the original draft. W-JX provided help on the revision on the manuscript. Y-NH and Z-YL provided help on the animal experiments. WH and C-SL provided help on the collecting of animal samples. X-MT provided funding to support the study. All authors contributed to the article and approved the submitted version.

## Footnotes

1
http://www.swisstargetprediction.ch

2
https://www.disgenet.org

3
https://www.genecards.org

4
https://string-db.org

5
https://david.ncifcrf.gov

6
https://www.rcsb.org

## References

[1] Richter B Hemmingsen B Metzendorf MI Takwoingi Y. Development of type 2 diabetes mellitus in people with intermediate hyperglycaemia. *Cochrane Datab Syst Rev.* (2018) 10:CD012661. 10.1002/14651858.CD012661.pub2 30371961 PMC6516891

[2] Zheng Y Ley SH Hu FB. Global aetiology and epidemiology of type 2 diabetes mellitus and its complications. *Nat Rev Endocrinol.* (2018) 2:88–98. 10.1038/nrendo.2017.151 29219149

[3] Inaishi J Saisho Y. Beta-cell mass in obesity and type 2 diabetes, and its relation to pancreas fat: a mini-review. *Nutrients.* (2020) 12:3846. 10.3390/nu12123846 33339276 PMC7766247

[4] Sun Z Sun X Li J Li Z Hu Q Li L Using probiotics for type 2 diabetes mellitus intervention: advances, questions, and potential. *Crit Rev Food Sci Nutr.* (2020) 4:670–83. 10.1080/10408398.2018.1547268 30632770

[5] Manukumar HM Shiva KJ Chandrasekhar B Raghava S Umesha S. Evidences for diabetes and insulin mimetic activity of medicinal plants: present status and future prospects. *Crit Rev Food Sci Nutr.* (2017) 12:2712–29. 10.1080/10408398.2016.1143446 26857927

[6] Luo K Huang W Qiao L Zhang X Yan D Ning Z Dendrocalamus latiflorus and its component rutin exhibit glucose-lowering activities by inhibiting hepatic glucose production via AKT activation. *Acta Pharm Sin B.* (2022) 5:2239–51. 10.1016/j.apsb.2021.11.017 35646547 PMC9136573

[7] Liu K Luo T Zhang Z Wang T Kou J Liu B Modified si-miao-san extract inhibits inflammatory response and modulates insulin sensitivity in hepatocytes through an IKKβ/IRS-1/Akt-dependent pathway. *J Ethnopharmacol.* (2011) 3:473–9. 10.1016/j.jep.2011.01.051 21296137

[8] Ma C Kang L Ren H Zhang D Kong L. Simiao pill ameliorates renal glomerular injury via increasing sirt1 expression and suppressing NF-κB/NLRP3 inflammasome activation in high fructose-fed rats. *J Ethnopharmacol.* (2015) 172:108–17. 10.1016/j.jep.2015.06.015 26117533

[9] Leem J Jung W Park HJ Kim K. A network pharmacology-based approach to explore mechanism of action of medicinal herbs for alopecia treatment. *Sci Rep.* (2022) 1:2852. 10.1038/s41598-022-06811-6 35181715 PMC8857194

[10] Yang L Wang H Song S Xu H Chen Y Tian S Systematic understanding of anti-aging effect of coenzyme q10 on oocyte through a network pharmacology approach. *Front Endocrinol.* (2022) 13:813772. 10.3389/fendo.2022.813772 35222272 PMC8874996

[11] Chen YM Lian CF Sun QW Wang TT Liu YY Ye J Ramulus mori (sangzhi) alkaloids alleviate high-fat diet-induced obesity and nonalcoholic fatty liver disease in mice. *Antioxidants.* (2022) 5:905. 10.3390/antiox11050905 35624769 PMC9137915

[12] Hasanvand A. The role of AMPK-dependent pathways in cellular and molecular mechanisms of metformin: a new perspective for treatment and prevention of diseases. *Inflammopharmacology.* (2022) 3:775–88. 10.1007/s10787-022-00980-6 35419709 PMC9007580

[13] Wang S Lu A Zhang L Shen M Xu T Zhan W Extraction and purification of pumpkin polysaccharides and their hypoglycemic effect. *Int J Biol Macromol.* (2017) 98:182–7. 28153462 10.1016/j.ijbiomac.2017.01.114

[14] Lopez-Vallejo F Caulfield T Martinez-Mayorga K Giulianotti MA Nefzi A Houghten RA Integrating virtual screening and combinatorial chemistry for accelerated drug discovery. *Comb Chem High Throughput Screen.* (2011) 6:475–87. 10.2174/138620711795767866 21521151

[15] Wang L Zhao Q Zhang Y Xue R Li S Li Y Network pharmacology and pharmacological evaluation for deciphering novel indication of sishen wan in insomnia treatment. *Phytomedicine.* (2022) 108:154500. 10.1016/j.phymed.2022.154500 36288650

[16] Ma Y Hu J Song C Li P Cheng Y Wang Y Er-xian decoction attenuates ovariectomy-induced osteoporosis by modulating fatty acid metabolism and IGF1/PI3K/AKT signaling pathway. *J Ethnopharmacol.* (2022) 301:115835. 10.1016/j.jep.2022.115835 36252878

[17] Xia T Liu CS Hu YN Luo ZY Chen FL Yuan LX Coix seed polysaccharides alleviate type 2 diabetes mellitus via gut microbiota-derived short-chain fatty acids activation of IGF1/PI3K/AKT signaling. *Food Res Int.* (2021) 150:110717. 10.1016/j.foodres.2021.110717 34865748

[18] Cao J Zheng R Chang X Zhao Y Zhang D Gao M Cyclocarya paliurus triterpenoids suppress hepatic gluconeogenesis via AMPK-mediated cAMP/PKA/CREB pathway. *Phytomedicine.* (2022) 102:154175. 10.1016/j.phymed.2022.154175 35609386

[19] Chen W Lin T He Q Yang P Zhang G Huang F Study on the potential active components and molecular mechanism of xiao huoluo pills in the treatment of cartilage degeneration of knee osteoarthritis based on bioinformatics analysis and molecular docking technology. *J Orthop Surg Res.* (2021) 16:460. 10.1186/s13018-021-02552-w 34273999 PMC8285844

[20] Chu S Zhang F Wang H Xie L Chen Z Zeng W Aqueous extract of guava (*Psidium guajava* L.) leaf ameliorates hyperglycemia by promoting hepatic glycogen synthesis and modulating gut microbiota. *Front Pharmacol.* (2022) 13:907702. 10.3389/fphar.2022.907702 35721172 PMC9198539

[21] Duan Y Dai H An Y Cheng L Shi L Lv Y Mulberry leaf flavonoids inhibit liver inflammation in type 2 diabetes rats by regulating TLR4/MyD88/NF-κB signaling pathway. *Evid Based Compl. Alt.* (2022) 2022:3354062. 10.1155/2022/3354062 35845591 PMC9279020

[22] Zhang Y Yan LS Ding Y Cheng B Luo G Kong J Edgeworthia gardneri (Wall.) meisn. Water extract ameliorates palmitate induced insulin resistance by regulating IRS1/GSK3beta/FoxO1 signaling pathway in human HepG2 hepatocytes. *Front Pharmacol.* (2019) 10:1666. 10.3389/fphar.2019.01666 32082162 PMC7002394

[23] Andersson C Vasan R. Epidemiology of cardiovascular disease in young individuals. *Nat Rev Cardiol.* (2018) 15:230–40. 10.1038/nrcardio.2017.154 29022571

[24] Hu N Zhang Q Wang H Yang X Jiang Y Chen R Comparative evaluation of the effect of metformin and insulin on gut microbiota and metabolome profiles of type 2 diabetic rats induced by the combination of streptozotocin and high-fat diet. *Front Pharmacol.* (2022) 12:794103. 10.3389/fphar.2021.794103 35046817 PMC8762251

[25] Cesur S Cam M Sayın F Su S Harker A Edirisinghe M Metformin-loaded polymer-based microbubbles/nanoparticles generated for the treatment of type 2 diabetes mellitus. *Langmuir.* (2022) 17:5040–51. 10.1021/acs.langmuir.1c00587 34096296

[26] Yang K Zhang S Geng Y Tian B Cai M Guan R Anti-inflammatory properties in vitro and hypoglycaemic effects of phenolics from cultivated fruit body of phellinus baumii in type 2 diabetic mice. *Molecules.* (2021) 8:2285. 10.3390/molecules26082285 33920885 PMC8071318

[27] Metz HE Kargl J Busch SE Kim KH Kurland BF Abberbock SR Insulin receptor substrate-1 deficiency drives a proinflammatory phenotype in KRAS mutant lung adenocarcinoma. *Proc Natl Acad Sci USA.* (2016) 31:8795–800. 10.1073/pnas.1601989113 27439864 PMC4978299

[28] Cui X Qian DW Jiang S Shang EX Zhu ZH Duan JA. Scutellariae radix and coptidis rhizoma improve glucose and lipid metabolism in T2DM rats via regulation of the metabolic profiling and MAPK/PI3K/Akt signaling pathway. *Int J Mol Sci.* (2018) 11:3634. 10.3390/ijms19113634 30453687 PMC6274950

[29] Chu Y Gomez RL Huang P Wang Z Xu Y Yao X Liver med23 ablation improves glucose and lipid metabolism through modulating FOXO1 activity. *Cell Res.* (2014) 10:1250–65. 10.1038/cr.2014.120 25223702 PMC4185346

[30] Li Y Ma Z Jiang S Hu W Li T Di S A global perspective on FOXO1 in lipid metabolism and lipid-related diseases. *Prog Lipid Res.* (2017) 66:42–9. 10.1016/j.plipres.2017.04.002 28392404

[31] Dong XC Copps KD Guo S Li Y Kollipara R Depinho RA Inactivation of hepatic foxo1 by insulin signaling is required for adaptive nutrient homeostasis and endocrine growth regulation. *Cell Metab.* (2008) 1:65–76. 10.1016/j.cmet.2008.06.006 18590693 PMC2929667

[32] Munekata K Sakamoto K. Forkhead transcription factor foxo1 is essential for adipocyte differentiation. *Vitro Cell Dev Biol Anim.* (2009) 10:642–51. 10.1007/s11626-009-9230-5 19585174

[33] Lin HV Accili D. Hormonal regulation of hepatic glucose production in health and disease. *Cell Metab.* (2011) 1:9–19. 10.1016/j.cmet.2011.06.003 21723500 PMC3131084

[34] Wu F Shao Q Xia Q Hu M Zhao Y Wang D A bioinformatics and transcriptomics based investigation reveals an inhibitory role of huanglian-renshen-decoction on hepatic glucose production of T2DM mice via PI3K/Akt/FoxO1 signaling pathway. *Phytomedicine.* (2021) 83:153487. 10.1016/j.phymed.2021.153487 33636476

[35] Pajvani UB Accili D. The new biology of diabetes. *Diabetologia.* (2015) 11:2459–68. 10.1007/s00125-015-3722-5 26248647 PMC4591190

[36] Wang Y Peng S Mei Z Jin C Kang J Xiang M Chlorogenic acid inhibits forming of diabetes mellitus in rats induced by high-fat high-sucrose and streptozotocin. *Pak J Pharm Sci.* (2020) 3:1063–72. 33191230

[37] Yadav A Singh A Phogat J Dahuja A Dabur R. Magnoflorine prevent the skeletal muscle atrophy via Akt/mTOR/FoxO signal pathway and increase slow-MyHC production in streptozotocin-induced diabetic rats. *J Ethnopharmacol.* (2021) 267:113510. 10.1016/j.jep.2020.113510 33141056

[38] Abdul-Ghani MA Tripathy D Defronzo RA. Contributions of β-cell dysfunction and insulin resistance to the pathogenesis of impaired glucose tolerance and impaired fasting glucose. *Diab Care.* (2006) 5:1130–9. 10.2337/dc05-2179 16644654

[39] Lv X Zhao Y Yang X Han H Ge Y Zhang M Berberine potentiates insulin secretion and prevents beta-cell dysfunction through the miR-204/SIRT1 signaling pathway. *Front Pharmacol.* (2021) 12:720866. 10.3389/fphar.2021.720866 34630099 PMC8493072

[40] Zhang H Wei J Xue R Wu JD Zhao W Wang ZZ Berberine lowers blood glucose in type 2 diabetes mellitus patients through increasing insulin receptor expression. *Metabolism.* (2010) 2:285–92. 10.1016/j.metabol.2009.07.029 19800084

[41] Gong J Hu M Huang Z Fang K Wang D Chen Q Berberine attenuates intestinal mucosal barrier dysfunction in type 2 diabetic rats. *Front Pharmacol.* (2017) 8:42. 10.3389/fphar.2017.00042 28217099 PMC5290458

[42] Zhao JD Li Y Sun M Yu CJ Li JY Wang SH Effect of berberine on hyperglycaemia and gut microbiota composition in type 2 diabetic goto-kakizaki rats. *World J Gastroenterol.* (2021) 8:708–24. 10.3748/wjg.v27.i8.708 33716449 PMC7934002

[43] Yao Y Chen H Yan L Wang W Wang D. Berberine alleviates type 2 diabetic symptoms by altering gut microbiota and reducing aromatic amino acids. *Biomed Pharmacother.* (2020) 131:110669. 10.1016/j.biopha.2020.110669 32937246

[44] Zhou H Jiang T Wang Z Ren S Zhao X Wu W Screening for potential -alpha -glucosidase inhibitors in coptis chinensis franch extract using ultrafiltration LC-ESI-MSn. *Pak J Pharm Sci.* (2014) 6:2007–12. 25410064

